# Intimate partner violence influences modern family planning use among married women in Tanzania: cross-sectional study

**DOI:** 10.1186/s12889-024-17666-z

**Published:** 2024-02-09

**Authors:** Mrimi S. Baritwa, Angelina A. Joho

**Affiliations:** Department of Clinical Nursing, School of Nursing and Public Health, The University of Dodoma, Dodoma, Tanzania

**Keywords:** Child spacing, Contraceptives, Pregnancy, Abuse, Violence, Family planning, Reproductive coercion

## Abstract

**Background:**

Married women who experience intimate partner violence (IPV) are less likely to negotiate with their partners on modern family planning (FP) use. This study aimed to determine the influence of intimate partner violence and sociodemographics on modern family planning use.

**Methods:**

A community-based cross-sectional study was conducted in the Mara region, Tanzania from April to July 2020. A total of 366 married women were interviewed. Data were collected using a structured interviewer-administered questionnaire. Analysis was done using SPSS version 25, and a binary logistic regression model was used to determine the predictors of modern FP use. The significance level was set at a p-value less than 0.05.

**Results:**

The overall prevalence of IPV was 73% with 54.1% physical, 36.3% psychological, and 25.4%, sexual violence. The prevalence of modern FP use was 62%, and the most (49.1%) common method practiced by married women was injection (Depo Provera). Physical violence (AOR = 0.32, *p* = 0.0056), and psychological violence (AOR = 0.22, *p* = 0.0022) had significantly reduced odds of modern FP use. Religion (AOR = 4.6, *p* = 0.0085), and availability of preferred modern FP methods (AOR = 9.27, *p* < 0.0001) had significantly increased odds of modern FP use.

**Conclusion:**

In this study, there is a positive association between the use of modern FP methods and IPV. To prevent IPV and its negative health consequences, it is crucial to involve community leaders and primary healthcare workers. They can help in identifying the best strategies to prevent IPV and promote the use of modern FP methods. It is equally important to involve male partners in reproductive health decisions, including the use of modern FP methods. This approach will help reduce reproductive coercion.

## Introduction

The use of modern family planning (FP) is a crucial intervention to prevent maternal deaths. It enables families to make informed decisions about the number of children they want and when to have them. Modern FP has immense benefits for individuals, families, society, and the community at large. It provides women with the time to engage in social roles, education, and vocational development, and empowers them [1]. It also allows for better care of existing children and reduces the number of high-risk births, unwanted pregnancies, unsafe abortions, poverty, and pregnancy-related complications [2–4].

Despite the well-known benefits of adhering to modern FP methods, the utilization of modern FP methods remains low in Tanzania. According to a recent Tanzania demographic and health and malaria indicator survey (TDHS-MIS) of 2022, reported that only 31% of married women in Tanzania use any form of modern FP method [5]. Similarly, low use of modern FP methods has been reported in other African countries, such as in Ethiopia 32.3% [6], Metekel Zone North West Ethiopia 18.6% [7], Nigeria 45.6% [8], and Zambia 21% [9].

The most affected are people living in the rural area [10]. In addition, low use of modern FP methods has been reported among married women with low self-efficacy, maternal low education levels, religious affiliation, low family income, partner’s low educational level, high number of living children, and women’s fertility preferences [10–13]. Women who experience intimate partner violence (IPV), partner disapproval of using modern FP methods, and other forms of reproductive coercion are also less likely to use modern FP methods [12–16]. This is particularly true for married women in the Mara region, where male partners are more involved in the decisions related to reproductive health and the use of FP. Despite the government’s efforts to raise awareness about modern FP methods, provide them free of charge, increase health facilities, and offer in-service training [17], the use of modern FP methods remains low among the affected population.

Studies have shown that IPV is a contributing factor that prevents married women from being able to discuss modern FP options with their male partner, which impairs their ability to make health-related decisions both physically and psychologically [12]. In addition, IPV also hinders female reproductive autonomy and is linked to early childbearing, high parity, and unintended pregnancy [14]. Reproductive coercion (RC) often accompanies IPV, where the husband and/or family members restrict the wives’ access and use of modern FP [14, 18]. Furthermore, husbands may physically harm their wives if they do not agree to become pregnant [19].

Despite the government’s efforts to reduce IPV by strengthening the police, legal, and health services and expanding modern FP services through increased numbers of health facilities and in-service training for healthcare workers, IPV and FP use continue to be major health challenges in the Mara region.

Therefore, this study aims to determine the impact of IPV and sociodemographic factors on modern FP use among married women in the Mara region.

### Conceptual framework

**Fig. 1:**
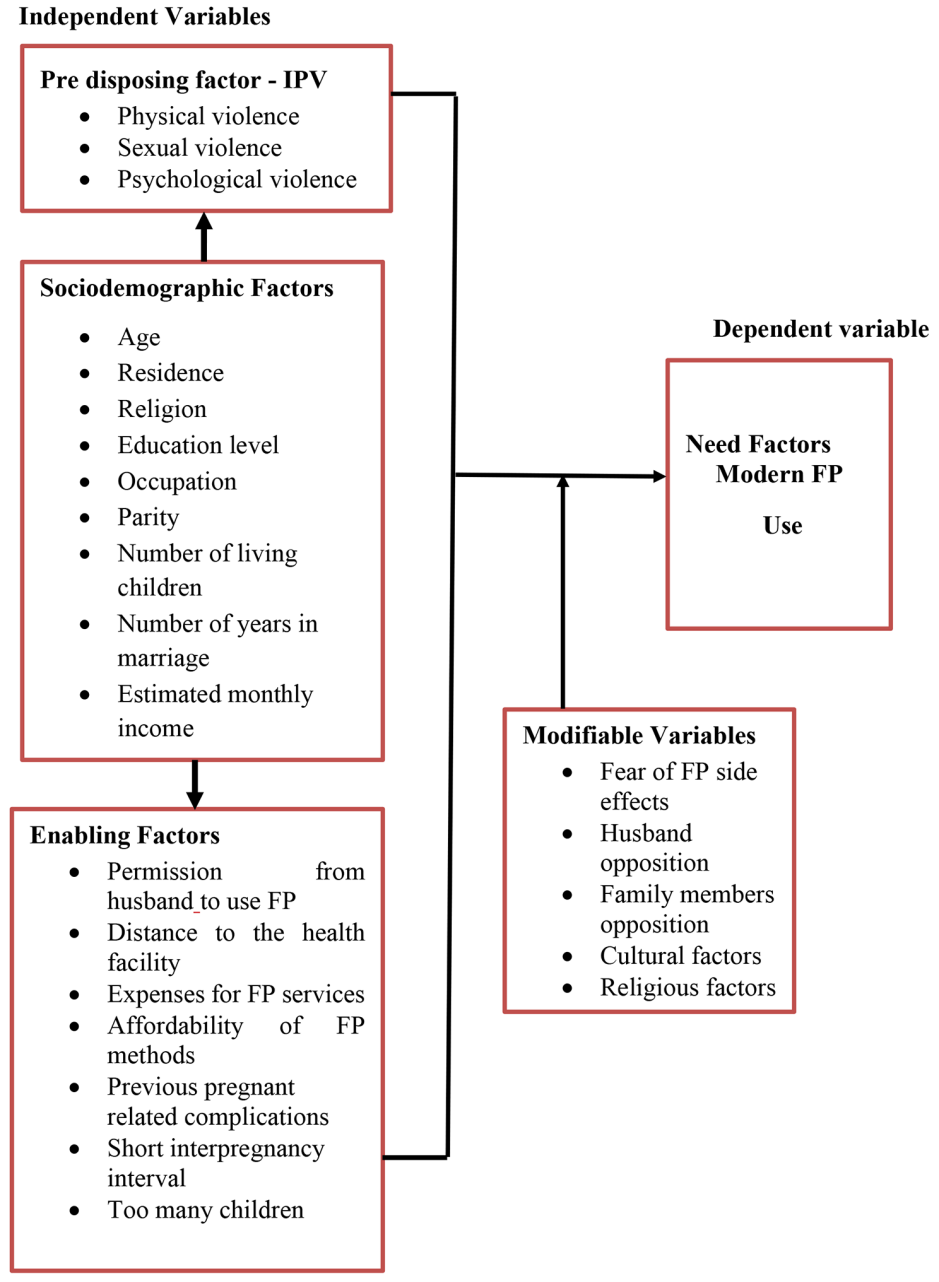
Conceptual framework for modern family planning use (Adopted from Anderson’s behavioral model, 1995)

In this study, we used Anderson’s behavioral model of health services to determine the relationship between intimate partner violence (IPV) and the use of modern family planning (FP) methods among married women. The study focuses on how IPV affects modern FP use. Anderson’s model considers access to modern FP methods as a result of individual decisions, in this case, the decisions of married women, and the availability of health care services (modern FP methods). The model has three categories: predisposing factors, enabling factors, and healthcare needs. Enabling factors include sociodemographic variables such as age, education level, economic status, and marital status. Predisposing factors include IPV, and the need factor is the uptake of modern FP methods as a dependent variable. Figure 1 shows how the variables interact to reflect the association between IPV and modern FP use. For example, the presence of IPV practices can deter married women from using modern FP methods, and sociodemographic factors can either strengthen or weaken the association. Intermediate variables can also contribute to strengthening or weakening the association. Enabling or need factors and predisposing factors can influence the use of modern FP methods among married women.

## Materials and methods

### Study design and setting

This study was a community-based cross-sectional study conducted in the Mara region, Tanzania that was conducted from April to July 2020. According to the TDHS-MIS report of 2022, the unmet need for modern FP methods among married women was 19.3%, with the prevalence of modern FP use being 28.8%, also, from the same report, it was reported that the fertility rate in the Mara region was 24.3% [5]. In the Mara region, the majority (80%) of married women reported experiencing at least one form of IPV as cited in a paper done in rural Tanzania [20]. Therefore, due to the low use of the modern FP method and the high prevalence of IPV, we wanted to know the influence of IPV and modern family planning use.

### Study population

This study included consented married and/or cohabiting women aged 15–49 years living in the Mara region for at least one year.

### Sample size determination

The sample size of 366 married women was calculated using the Cochran formula (1977) in which 61% proportional to modern FP use [3], and a permissible marginal error of 5% and constant standard normal variation of 1.96 at 95% confidence interval were applied in calculating the sample size.

### Sampling technique

In this study, a range of sampling techniques was used to select the study settings and the population. The Mara region was selected purposively because of its low (19.3%) unmet need for modern FP usage [5] and the highest rate (78%) of IPV practice [13]. The region has seven districts, out of them four districts were randomly selected using simple random sampling, and one division was selected in each selected district. From each selected division, two wards were selected two villages were selected in each selected ward, and households were randomly selected from each village. The interviews were conducted with married and/or cohabiting women living in these households using criterion sampling.

### Data collection tool and procedure

#### Data collection tool

To ensure the study’s reliability, a standardized interviewer-administered questionnaire was adopted from the previous study [21]. Regarding IPV questionnaire was adopted from a Revised Conflict Tactics Scale 2 (CTS-2) developed Straus et al. (1996) with Alpha values of 0.86, 0.87, and 0.79 for physical violence, sexual violence, and psychological violence, respectively. The questionnaire was in English, translated into Kiswahili language (the national language spoken in Tanzania), and then back-translated to English for consistency.

### Data collection procedure

The data were collected through a structured questionnaire by a team of four highly trained female research assistants. All of them were nurses with counseling expertise and fluency in the local language of the study area. The research assistants, along with the principal investigator, were responsible for ensuring that the data collection procedures were carried out correctly. To ensure confidentiality, interviews were conducted in a secluded area to avoid being overheard by other household members. In some houses where husbands were available were asked permission for their wives to participate in the study and they were also asked to provide a room for wives to be free to talk. The principal investigator ensured that all selected households were contacted and all married and/or cohabiting women were interviewed. In case, the eligible participant was not at home, the research assistant waited for her to come or come back later at the convenience early time. The principal investigator was responsible for making sure that all questionnaires were collected and filled in before the team left the village.

### Measurements of variables

Intimate partner violence (physical, sexual, and psychological) was measured using Conflict Tactics Scale version 2 (CTS-2) which consisted of 12 items [22]. The tool was made up of seven categories; 1 if IPV occurred once in the past 1 year, 2 if occurred twice in the past 1 year, 3 if occurred 3–5 times in the past 1 year with 4 midpoints, 4 if occurred 6–10 times in the past 1 year with 8 midpoints, 5 if occurred 11–20 times in the past 1 year with 15 midpoints, 6 if occurred more than 20 times in the past 1 year with recommended 25 midpoint, 7 if not in the past 1 year, but happened before and 0 if never happened. A woman was classified as having experienced IPV if she responded affirmatively to one or more of the questions relating to specific IPV forms [23].

Questions used for the assessment of IVP forms: **Physical violence** included seven dimensions questions: [1] push you, shake you, or throw something at you [2]? slap you [3]? twist your arm or pull your hair [4]? punch you with his/her fist or with something that could hurt you [5]? kick you, drag you, or beat you up [6]? try to choke you or burn you on purpose [7]? threaten or attack you with a knife, gun, or any other weapon? **Sexual violence** experience included four dimensions questions: [1] physically force you to have sexual intercourse with him even when you did not want to [2]? physically force you to perform any other sexual acts against your will [3]? force you with threats or in any other way to perform sexual acts you did not want to? Forcing touched your.

Body against your will? **Psychological violence** experience included 11 questions: [1] Threatened to harm or hurt you [2]? Refused to talk with you [3]? Controlling your behavior [4]? Control any source of your income [5]? Criticize you in public or intimidate/undermine you [6]. insult you or make you feel bad about yourself or harass/command you [7]? Defamation/abusive attack [8]? Say or do something to humiliate you in front of others [9]? Insulted your valued beliefs [10]? Insulted your religion [11]? Threatened you to leave from marriage?

Modern FP use was measured into two categories; with 1 representing a woman using any modern method of contraception and 0 for those not using any method of contraception. The modern contraceptive methods included in this study were: oral pills, injectables, implants/Norplant, intrauterine contraceptive devices (IUDs), male condoms, and sterilization) [24].

### Data analysis

Statistical Package for Social Sciences (SPSS) software, version 25.0 was used for data analysis. Categorical variables were presented in proportions. Mean and standard deviation were computed for continuous variables before data categorization. The association of the categorical variables was determined using the Chi-Square test, and all variables with *p* ≤ 0.2 were taken to regression analysis. Multivariable analysis under binary logistic regression analysis was used to determine the predictors for IPV by calculating the adjusted odds ratios (AORs) at a 95% confidence interval (CI). A two-tailed *p* < 0.05 was considered statistically significant.

Multivariable analysis under binary logistic regression analysis was used to determine the predictors for knowledge, attitude, and practice by calculating the adjusted odds ratios (AORs) at a 95% confidence interval (CI). A two-tailed *p* < 0.05 was considered statistically significant.

## Results

### Sociodemographic characteristics of respondents

A total of 366 married women included in this study were aged between 15 and 49 years with a mean age of 30.27 ± 7.102 years and the majority 272 (74.3%) were from rural areas. Regarding religion the majority of participants 254 (69.4%) were Christians. More than half of the respondents had attained primary school education 197 (53.8%). Over half 191 (52.2%) of respondents were unemployed. Most of them 166 (45.4%) reported having delivered three to four times, of them 137 (37.4%) had 6–10 years of marriage (Table 1).

**Table 1:** Sociodemographic characteristics of the respondents (*N* = 366)

Variable	Frequency (n)	Percentage (%)	Mean ± SD
**Age (years)**			30.27 ± 7.102
15–24	85	23.2	
25–34	173	47.3	
35–44	90	24.6	
45–49	18	4.9	
**Residence**			
Urban	94	25.7	
Rural	272	74.3	
**Religion**			
No religion	61	16.7	
Christian	254	69.4	
Muslim	51	13.9	
**Highest education level**			
Informal education	60	16.4	
Primary education	197	53.8	
Secondary education	72	19.7	
College/university	37	10.1	
**Occupation**			
Employed	26	7.1	
Self-employed	149	40.7	
Unemployed	191	52.2	
**Number of pregnancies**			4.17 ± 1.417
0	12	3.3	
1–2	101	27.6	
3–4	166	45.4	
5+	87	23.8	
**Number of living children**			3.73 ± 1.468
0	27	7.4	
1–2	133	36.3	
3–4	148	40.4	
5+	58	15.9	
**Duration of years in marriage**			5.12 ± 0.783
< 5	93	25.4	
6–10	137	37.4	
> 11	136	37.2	
**Monthly income average (TZS)**			
< 50,000	219	59.8	
50,000–100,000	80	21.9	
˃100,000	67	18.3	

### Prevalence of intimate partner violence and modern family planning use

**Fig. 2:**
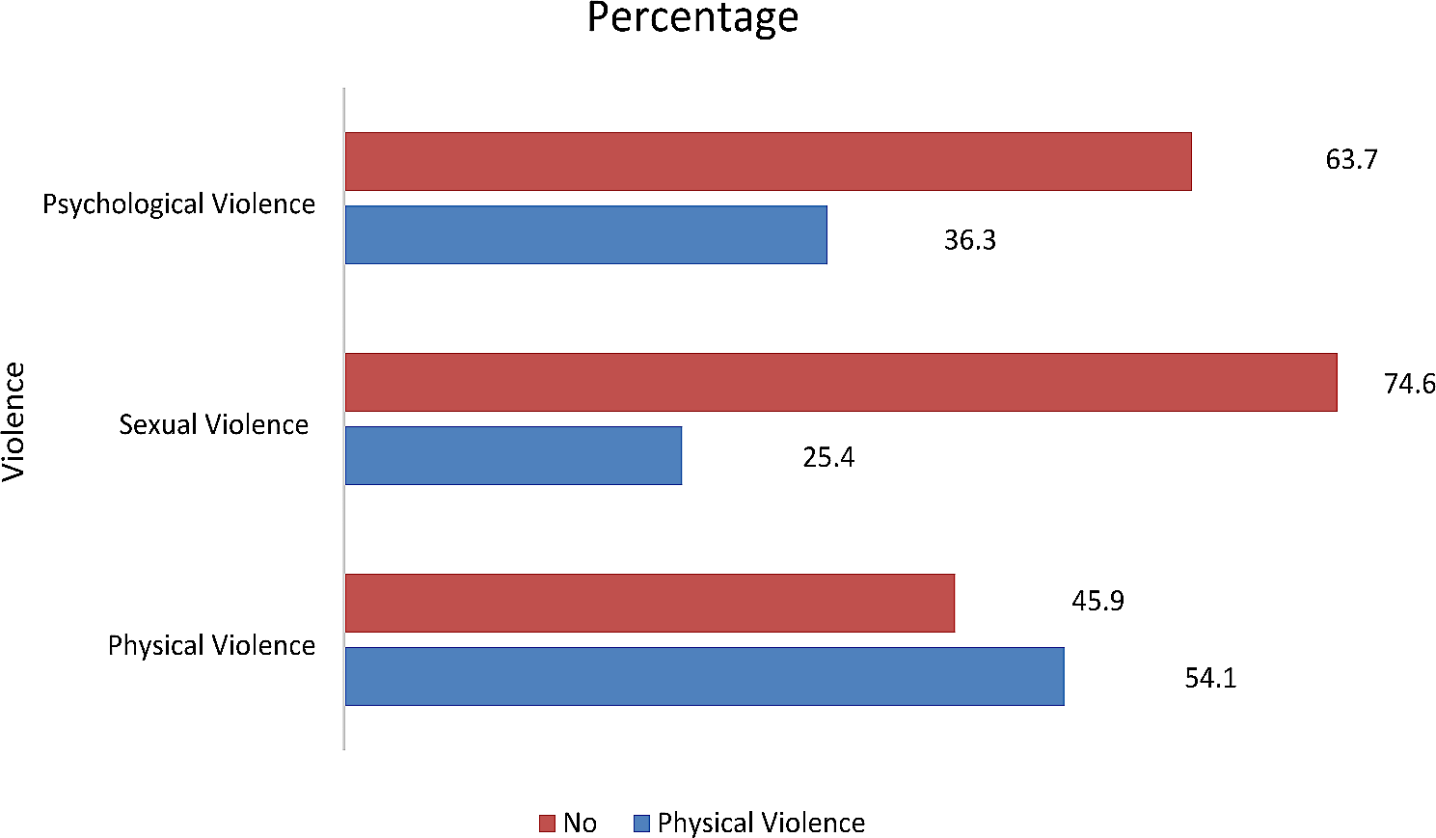
Prevalence of intimate partner violence

The prevalence of intimate partner violence was 73.2%, with 54.1%, 25.4% and 36.3% being physical, sexual, and psychological IPV, respectively (Fig. 2). The prevalence of modern FP use among married women was 62.02%.

### Types of modern family planning methods used

**Fig. 3:**
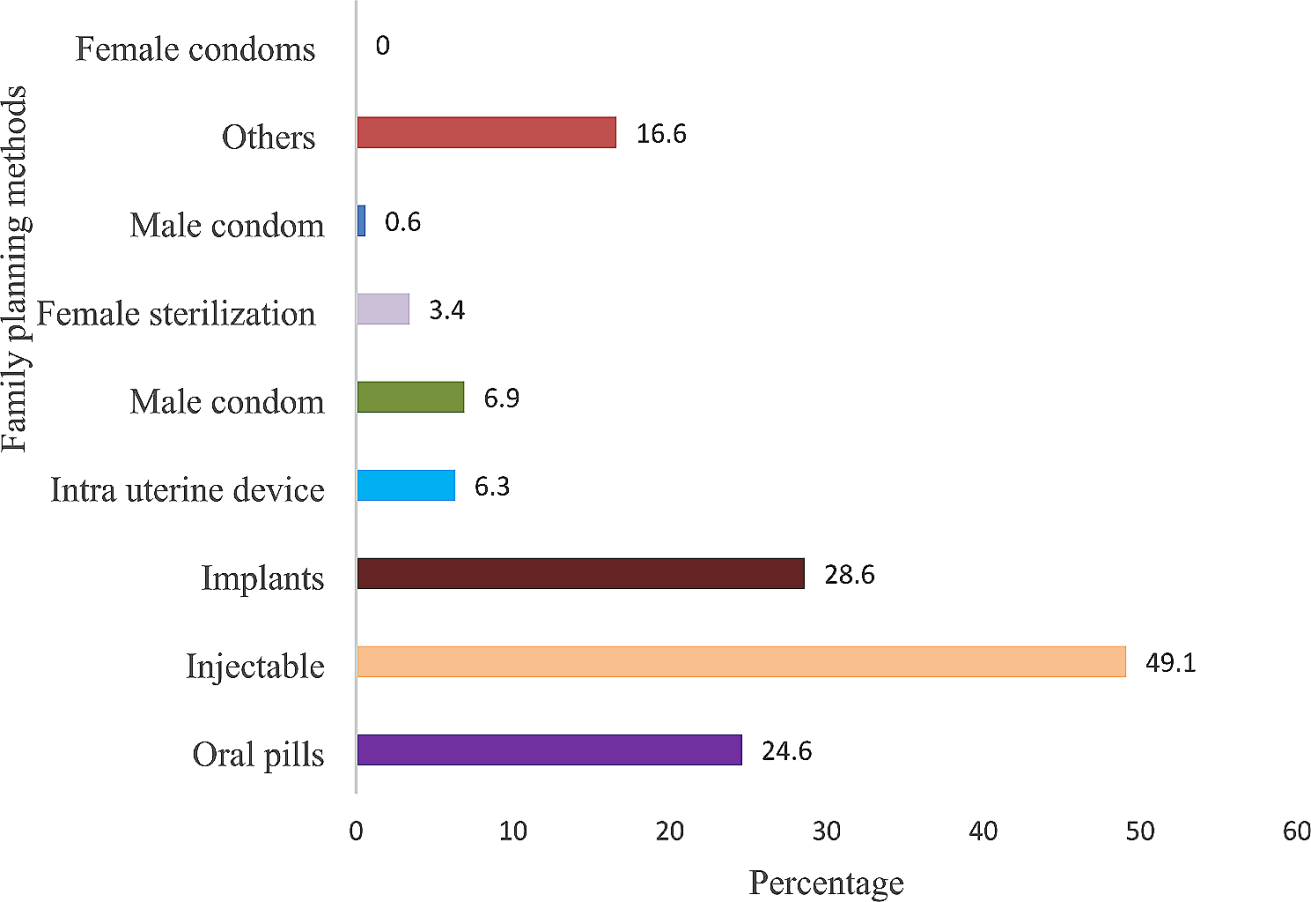
Types of modern FP methods used among married women

The most common family planning method used was injectable 49.1%, and the last method used was male sterilization 0.6% (Fig. 3).

### Reported barriers to modern family planning use

**Fig. 4:**
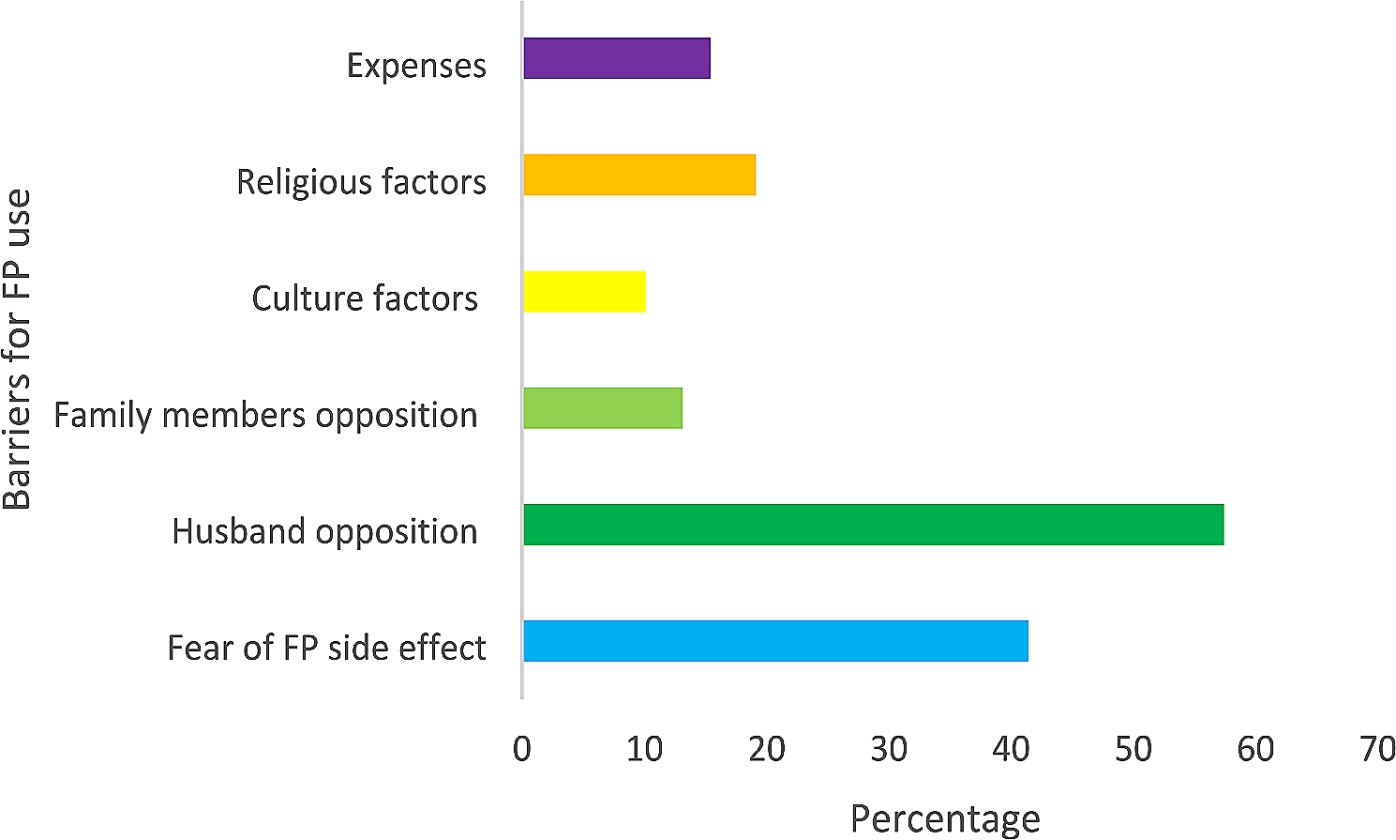
Reported barriers to modern FP use among married women

Among the barriers to modern family planning use in this study, 57.4% were due to husband opposition followed by 41.4% resulting from fear of family planning side effects. The least barrier to using FP among the participants was cultural factors which accounted for 10% (Fig. 4).

### Proportional exposure to intimate partner violence by age

Women aged 25–34 years were most affected by all forms of IPV with overall prevalence of physical, sexual, and psychological IPV were 54.9%, 28.3%, and 32.4%, respectively. Those aged 45 years and above were less commonly abused with all three forms of IPV physical, sexual, and psychological (55.6%, 27.8%, and 27.8%, respectively) (Table 2).

**Table 2:** Proportional exposure to intimate partner violence by age (*N* = 366)

Age category	Physical violence		Sexual violence		Psychological violence	
	No n (%)	Yes n (%)	No n (%)	Yes n (%)	No n (%)	Yes n (%)
15–24	36 (42.4)	49 (57.7)	71 (83.5)	14 (16.5)	51 (60.0)	34 (40.0)
25–34	78 (45.1)	95 (54.9)	124 (71.7)	49 (28.3)	117 (67.6)	56 (32.4)
35–44	46 (51.1)	44 (48.9)	65 (72.2)	25 (27.8)	52 (57.8)	38 (42.2)
≥ 45	8 (44.4)	10 (55.6)	13 (72.2)	5 (27.8)	13 (72.2)	5 (27.8)

### Factors influencing modern FP use among married women

After adjusting for all factors physical violence, psychological violence, religion, and availability of FP remained significantly associated with FP use. Married women who experienced physical violence were 68% less likely to use FP compared to those who were not experiencing physical violence (AOR = 0.32, 95% CI: 0.29–3.82, *p* = 0.006). Those who experienced psychological violence were 78% less likely to use FP (AOR = 0.22, 95% CI: 0.08–0.58, *p* = 0.002). The odds of using FP were almost 5 times greater among Christian married women compared to women who were non-religious (AOR = 4.61, 95% CI: 1.48–14.41, *p* = 0.009). Likewise, Muslim married women were almost 3 times more likely to use FP compared to non-religious married women (AOR = 2.70, 95% CI: 0.61–12.01, *p* < 0.000).

Also, the odds of using FP methods were 9 times greater among married women who agreed that FP methods were available compared to those who claimed FP methods were not available (AOR = 9.27, 95% CI: 7.15–84.49, *p* < 0.000). Married women who reported that FP is expensive were almost 2 times more likely not to use the FP methods compared to their counterparts (AOR = 1.7, 95% CI: 0.34–8.2, *p* = 0.525). Those who had a fear of side effects had 55% less chance of using FP methods (AOR = 0.45, 95% CI: 0.19–1.10, *p* = 0.088). Likewise, women who experienced husband opposition to using FP methods were 1.4 times more likely not to use FP than those who did not (AOR = 1.44, 95% CI: 0.61–3.40, *p* = 0.412). Those experiencing religious disapproval of using FP methods were 63% less likely to use FP methods compared to those who were not experiencing religious opposition (AOR = 0.37, 95% CI: 0.13–1.04, *p* = 0.058) (Table 3).

**Table 3:** The association between IPV, other factors, and modern FP use (*N* = 366). FP = Family plan, IPV = intimate partner violence, OR = Odds ratio, AOR = Adjusted odds ratio, CI = Confidence interval

Variable	OR	95% CI		p-value	AOR	95% CI		p-value
		Lower	Upper			Lower	Upper	
Physical violence								
No	1				1			
Yes	0.60	0.39	0.92	**0.020**	0.32	0.29	3.82	**0.006**
Sexual violence								
No	1				1			
Yes	0.67	0.42	1.08	0.099	1.18	0.42	3.30	0.756
Psychological violence								
No	1				1			
Yes	0.57	0.37	0.88	**0.001**	0.22	0.08	0.58	**0.002**
Religion								
No religion	1				1			
Christian	2.02	1.15	3.55	**0.015**	4.61	1.48	14.41	**0.009**
Muslim	1.36	0.65	2.88	0.418	2.70	0.61	12.01	0.192
Expenses to purchase FP								
Yes	11.65	2.75	49.34	**0.001**	1.68	0.34	8.19	0.525
No	1				1			
Availability of FP								
Yes	8.50	5.13	48.63	**0.000**	9.27	7.15	84.49	**0.000**
No	1				1			
Pregnancy complications								
Yes	3.44	1.48	7.97	**0.004**	3.28	0.64	16.79	0.154
No	1				1			
Fear of FP side effects								
Yes	0.48	0.31	0.73	**0.001**	0.45	0.19	1.10	0.079
No	1				1			
Husband opposition								
Yes	0.67	0.44	1.04	**0.073**	1.44	0.61	3.40	0.412
No	1				1			
Religious disapproval								
Yes	0.41	0.24	0.69	**0.001**	0.37	0.13	1.04	0.058
No	1				1			

## Discussion

Intimate partner violence (IPV) is linked to reproductive coercion, which acts as a major barrier to women’s reproductive autonomy. This includes their use of modern FP methods, especially among married women. IPV poses a significant barrier for young married women to make informed decisions about their reproductive health services and use of FP.

The key findings of this study include the high prevalence of IPV and the level of modern FP use. Young married women aged 25–34 years were most affected by all forms of IPV. Modern FP use was negatively associated with physical and psychological violence, cost of accessing modern FP, side effects of FP, and husband opposition/disagreement of their wives to use modern FP. Moreover, modern FP use was positively associated with religion, and the availability of modern FP methods.

In the current study, the overall prevalence of IPV among married women in the region was 73.2% with physical violence (54.1%) being the most common form of reported IPV. The overall prevalence of IPV in the present study is higher compared to 26.2% in the study which was conducted in India [25], 20% in Ethiopia [18], 16% in Northern California [26], 32% in Rhode Island [27], and 24.8% in Nigeria [28]. The observed discrepancy may be due to differences in the socioeconomic status of married women and the nature of the study area. In the current study, the practice of IPV in the study area is considered normal behavior and socially accepted as biting a woman is a way of teaching discipline. Another reason is that most men dominate the relationships, and women are financially dependent on their male partners and are often unaware of their legal rights [29].

In the current study, more than half of married women reported having experienced physical violence when wanted to access and/or use FP. These findings are consistent with similar studies conducted in Bangladesh [30], rural India [31], Niger [32], and India (27 ), which also found that women who experienced physical intimate violence were at high risk of reproductive coercion. The violence against women interferes with their ability to access FP services, which increases their risk of unplanned pregnancy and unsafe abortion [14, 18, 19, 28, 33, 34]. Therefore, it is important to integrate reproductive coercion interventions into maternal, reproductive clinics to provide women with counseling and support. Additionally, male partners should be included in all discussions related to reproductive health, as they are often the perpetrators of IPV. Educating men about FP methods and other reproductive health services can help to reduce the incidence of IPV.

Our study found that the prevalence of IPV and modern FP use was relatively high, while at the same time, the rate of women experiencing reproductive coercion (physical violence and prohibition of women from accessing or/and using modern FP methods) is also high. This relationship indicates that women are practicing covert contraception that is using modern FP methods without their male partner’s knowledge [15, 35–39]. This situation puts women at high risk of IPV once their partners discover [12, 14, 31, 40], that is a possibility of reverse causality (using modern FP can also cause IPV). Studies have shown that married women who use modern FP without partner consent experience increased IPV once discovered by their husbands [12, 14, 31, 40]. Furthermore, studies reported that including male partners in the decision of modern FP utilization could reduce the incidence of unwanted pregnancies, increase the use of FP, and reduce the incidence of IPV [13, 40, 41]. Therefore, the essence of including male partners in modern FP education is of paramount importance.

The overall prevalence of modern FP use in the current study was 62.0%. This prevalence of modern FP use observed was similar to that of the national target of 60% by 2020 [13]. The prevalence of modern FP observed in our study is higher compared to reported in previous studies conducted in Guinea 51.2% [42], Ghana 21% [43], rural Northeastern Nigeria 26% [44], Kenya 54% [45], and in Pakistan 34% [25]. The difference in prevalence could be IPV and/or reproductive coercion among our study participants. We found that most women who experienced any form of IPV preferred to use injectable modern FP method and they used it without their husbands’ knowledge. This might be one way for women who tt experienced reproductive coercion to reduce the risk of unplanned pregnancy [32, 42].

In the current study, it was found that married women who had suffered physical and psychological violence were less likely to use modern FP methods as compared to non-violated women. Similarly, several other studies have also reported the same. For example, a study was conducted in India [12], Nepal [47], and rural Tanzania [40]. The reasons for not using modern FP among married women who were victims of IPV were multifaceted. Women are scared of being caught using modern FP methods, which could also put them at higher risk of increased IPV [40, 47]. Additionally, financial dependence on male partners. These women might be financially poor, which makes it difficult for them to access or buy modern FP [29, 48].

In this study, married women reported that the obstacle to using modern FP methods is fear of side effects. This is in line with several studies, For instance, in the study conducted in Ethiopia [49], Kenya [50], Kilifi in Kenya [51], Ethiopia [52], Uganda [53], in Kilimanjaro, Tanzania, Dar es Salaam, Tanzania [54]. This may be due to hearing from friends and families, misconceptions information [52, 53, 55], cultural ideology of having many children, lack of male involvement [55], and covert contraception [15, 35–39]. However, in the real ground, modern FP methods have many reported side effects such as irregular menstrual bleeding, amenorrhea or oligomenorrhea delayed return fertility [41, 56–58], headache, high blood pressure, and varicose vein [59]. For these reasons the majority of married women stop using modern FP methods, this reason may be caused by inadequate counseling, low level of education, and poor communication with health care providers [41, 54, 55]. Therefore, healthcare providers should provide education, counseling, and communication to embrace knowledge to users of modern FP methods and their partners.

Male partners who use reproductive coercion by prohibiting their wives from accessing and using FP methods were negatively associated with modern FP use, which put their wives at a higher risk of having unplanned pregnancies, less use of FP methods, social consequences, and poor health outcomes, including HIV infection [14, 16, 60]. Furthermore, IPV has an impact on physical and psychosocial health outcomes [39]. Partner’s lower level of education, preference to have children in the future, less number of live children, and husband’s approval were cited as reasons for not utilizing modern family planning [27]. The low use of FP was also reported to be associated with discouragement of using FP from an intimate partner [54]. Male dominance was reported as a significant factor in the low use of FP [33], of which men act as decision makers over the health of their wives on the use of FP which in turn could lead to unplanned pregnancies which is directly associated with reproductive coercion [18]. Another study conducted in 29 low and middle-income countries reported a similar finding that women’s experienced IPV were associated with increased odds of having an unintended pregnancy [61]. African men who prohibit their wives from using modern FP methods, need many children for them is proud, also these men think that using modern FP methods may cause infertility [41, 54, 55]. This also justifies the importance of male partner involvement in reproductive health services.

Religion was significantly associated with current modern contraceptive use. In this study, Christian married women were independent factors for using modern family planning methods than their counterparts. Similar findings were reported by several studies conducted elsewhere. Such as in the study conducted in rural Tanzania [62], Ethiopia [63], Nigeria [40], Rwanda [38] and Ethiopia [49]. The probable reason for the Christian religion being more likely to use modern FP methods could be the civilization on the importance of child spacing [64]. Likewise, it has been reported by the leaders of the Roman Catholic church that Catholics do not have to breed like rabbits, however, they are allowed to use temporal family planning methods and prohibited from using emergency contraception and abortion without exception, even in life-threatening of a pregnant woman [65]. Additionally, Protestantism has been reported to be flexible in using family planning for the sake of family size [65].

However, the findings of the current study contradict those reported by Radhika et al. [64], who found that Christian and Muslim women believed family planning was incompatible with their faith. These women insisted that it was their responsibility to give birth to as many children as God would give them [64]. Findings from another study also reported that men who oppose their wives using family planning cite religious beliefs to justify having more children such belief is God allows us to go and multiply [62]. An educational intervention focused on the importance of family planning should be specifically targeted toward religious leaders as they hold significant influence within the community. Once they understand the benefits of family planning, it will be easier for them to encourage their followers to use it [64].

The findings of the current study showed that respondents were more likely to utilize modern FP methods if the preferred FP methods were available in health facilities compared to those respondents who were not able to access the methods. Similar results were reported in the studies done in Ethiopia, Zambia, and Burundi [44–46].

### Limitations of study

This study has some limitations due to its cross-sectional study design, which does not allow us to establish causal relationships between variables. Furthermore, there is a possibility of recall bias that might increase or decrease the strength of the observed associations as participants were asked to recall past information. Additionally, the use of convenience sampling resulted in an unequal representation of patients, which may have led to selection bias.

### Conclusion and recommendations

Efforts to increase the use of modern FP methods should be integrated with women’s rights as it has been observed that all forms of violence have significantly reduced the use of modern FP methods. To ensure male partners are included in the decision to use modern FP, community involvement is necessary to educate them about the advantages of FP use. Further research is needed to investigate IPV, reproductive coercion, and modern FP use among married young/adolescent girls as this group might be at higher risk of reproductive coercion.

## Data Availability

The data related to this study are available upon request, provided such requests are reasonable. Due to ethical restrictions, the data underlying the study cannot be made public. However, it is possible to obtain access to the dataset by submitting a reasonable request to the Directorate of Research Publication and Consultancy (DRPC) at the University of Dodoma. The address for the DRPC is P.O. Box 259, Dodoma, Tanzania.drpc@udom.ac.tz.

## Abbreviations

DHIS: Demographic Health Survey
FP: Family planning
IUD: Intrauterine device
NBS: National Bureau of Statistics
UN: United Nations
